# Polymer Electrolyte Membranes

**DOI:** 10.3390/membranes11040244

**Published:** 2021-03-29

**Authors:** Byungchan Bae, Dukjoon Kim

**Affiliations:** 1 Fuel Cell Laboratory, Korea Institute of Energy Research, Daejoen 34129, Korea; 2 Department of Renewable Energy Engineering, University of Science and Technology, Daejeon 34113, Korea; 3 School of Chemical Engineering, Sungkyunkwan University, Suwon 440-746, Korea

Recently, polymer electrolyte membranes have been used in various electrochemical energy devices and other applications, such as fuel cells, lithium secondary batteries, redox flow batteries, electrodialysis, and membrane capacitive deionization. Since an electrolyte membrane greatly affects the performance and durability of a device, interest in the role of the electrolyte membrane has been increasing. The development of low-cost and high-performance electrolyte membranes has increased to replace expensive commercial electrolyte membranes. 

This Special Issue suggests various approaches in selecting ion-exchange membranes for fuel cells and secondary batteries. The original synthetic method and characterization of the anion exchange membranes have highlighted great insights for the development of high-performance alkaline membranes, as some of those membranes exhibited excellent ion conductivity and durability under severe alkaline conditions. The electrochemical performance of the alternative hydrocarbon membranes was comparable to that of the benchmark PFSA (Perfluorinated Sulfonic Acid). These approaches provided insight into overcoming the drawbacks of hydrocarbon membranes. Finally, a separator for secondary batteries was introduced to cover another field of ion-exchange membranes.

First, three papers related to the synthesis and characterization of anion exchange membranes were published. Tuan et al. [1] reported a novel anion exchange membrane via crosslinking of quaternized poly epichlorohydrin (QPECH) with 1-(3-aminopropyl) imidazole grafted poly(arylene ether ketone) (PAEK-API). Various membrane properties, such as anion conductivity, water uptake, length swelling percentage, and thermal- mechanical-chemical stabilities, were investigated. The QPECH/PAEK-API membrane showed a high hydroxide ion conductivity, from 0.022 S cm^−1^ (30 °C) to 0.033 S cm^−1^ (80 °C), and excellent mechanical strength, associated with a low water uptake of less than 40%, even at 80 °C. Furthermore, the QPECH/PAEK-API membranes showed thermal stability up to 250 °C and chemical stability for 30 days in a 4 NaOH solution, without significant loss of ion exchange capacity. The free imidazolium groups in the hydrated membranes would create ionic clusters with various dimensions, as shown by SAXS analysis, which would directly affect the membrane properties.

Lee et al. [2] reported producing anion exchange membranes using perfluorinated polymers containing quaternary ammonium groups prepared from Nafion- and Aquivion-based sulfonyl fluoride precursors by the Menshutkin reaction. Both perfluorinated polymer-based membranes exhibited distinct hydrophilic–hydrophobic phase-separated morphologies, resulting in high ion conductivity despite their low ion-exchange capacities and limited water uptake properties. Moreover, the capacitive deionization performance and stability of the perfluorinated polymer membranes were superior to those of the commercial Fumatech membrane. Although the perfluorinated polymers demonstrated high alkaline anion conductivities, their alkaline stabilities were poorer than those of the hydrocarbon-based Fumatech ionomer, owing to the strong electron-withdrawing characteristics of the perfluorinated structure. Consequently, the chemical structures of perfluorinated polymer-based anion exchange membranes require further study to improve their chemical stability and expand their application potential.

Mayadevi et al. [3] presented a series of poly(meta/para-terphenylene-methyl piperidinium)-based anion exchange membranes devoid of benzylic sites and aryl ether bonds for possible application. They reported, for the first time, the development of copolymers between para-terphenyl and meta-terphenyl units with different ratios to balance the conformational changes. The copolymers were composed of both linear para-terphenyl units and kink-structured meta-terphenyl units. The meta-connectivity in terphenyl units allowed the polymer backbones to fold back, maximizing the interactions among the hydrocarbon polymer chains and enhancing the peripheral formation of ion aggregates because of the free volume generated by the kink structure. The m-p-MP-50, with an IEC (Ion Exchange Capacity) of 2.49 meq/g, presented a high conductivity of 130.39 mS/cm at 80 °C, as well as good mechanical properties and high thermal and alkaline stability. The m-p-MP-50 also showed a peak power density of 172 mW/cm^2^ at a current density of 407 mA/cm^2^ and a temperature of 60 °C. These results are comparable to or better than those reported for other AEMs based on the rigid polymer backbones.

Furthermore, four other papers on cation exchange membranes have been published. Manohar et al. [4] reported an aromatic polymer (poly (1,4-phenylene ether-ether-sulfone)—SPEES interconnected and crosslinked with an aliphatic monomer (2-acrylamide-2-methyl-1-propane sulfonic; AMPS) with a sulfonic group to enhance the conductivity and an aliphatic chain of AMPS to make it flexible. This allowed for the optimization of the synthesis of polymer electrolyte membranes with tailor-made combinations of conductivity and stability. The AMPS-based membrane deteriorated at high temperatures (above 65 °C) because of the high functional charge of sulfonic, tertiary amine, and ketone. The prepared membranes showed improved proton conductivity up to 0.125 S cm^−1^, which is much higher than that of Nafion N115 (0.059 S cm^−1^). All these membranes have been shown to have constant conductivity at a stable temperature (45 °C). They can be of great benefit and cost-effectiveness at low temperatures and low RH (Relative Humidity) for fuel cell applications.

Sutradhar et al. [5] reported poly(benzophenone)s membranes (SI-PBP) containing acidic sulfonyl imide groups prepared from 2,5-dichlorobenzophenone (DCBP) monomers by C-C coupling polymerization using a Ni (0) catalyst. The entirely aromatic C-C coupled polymer backbones of the SI-PBP membranes provided exceptional dimensional stability with a rational ion exchange capacity (IEC) of 1.85 to 2.30 mS/cm. The as-synthesized SI-PBP 3 membranes exhibited higher proton conductivity (107.07 mS/cm) with a maximum power density of 0.638 W/cm^2^ than Nafion 211^®^ (104.5 mS/cm and 0.62 W/cm^2^). The notable thermal and chemical stabilities of the SI-PBP membranes were assessed by thermogravimetric analysis (TGA) and Fenton’s test, respectively. Moreover, the pendant sulfonyl imide groups provided well-defined ion-conducting channels for proton conduction throughout the polymer network. 

Wakiya et al. [6] reported novel nanofiber framework (NfF)-based composite membranes composed of phytic acid (Phy)-doped polybenzimidazole nanofibers (PBINf) and a Nafion matrix electrolyte. The NfF composite membrane showed higher proton conductivity and lower activation energy than the recast-Nafion membranes, especially at low relative humidity. The high proton mobility of NfF-CM3 at low water uptake compared to other membranes was also notable. Such outstanding characteristics were considered to originate from the continuously formed effective proton conductive pathway consisting of densely accumulated phosphoric acid and sulfonic acid groups at the interface of the nanofibers and the Nafion matrix.

Ahn et al. [7] synthesized CeOx hybrid nanoparticles and evaluated them for use as radical scavengers in place of commercially available Ce(NO_3_)_3_ and CeO_2_ nanoparticles. When CeOx hybrid nanoparticles were used for membrane formation, the resulting membranes exhibited improved proton conductivity (improvement level = 2–15% at 30–90 °C), and electrochemical single-cell performance –OH groups on the hybrid nanoparticles acted as proton conductors. The CeOx_A and CeOx_B maintained stable dispersion phases without any aggregation or precipitation, even in long-term storage for months. Despite the introduction of only a small amount (i.e., 1.7 mg/cm^3^), their antioxidant effect was sufficient to alleviate the radical-induced decomposition of perfluorinated sulfonic acid ionomer under Fenton test conditions and extend the chemical durability of the resulting reinforced membranes under fuel cell operating conditions. The CeOx hybrid nanoparticles developed in this study are expected to provide an effective avenue for the design of ultimate radical scavengers and their applications. 

Finally, a paper revealing a novel multilayer separator was published for lithium-ion batteries (LIBs). Bui et al. [8] developed a novel multilayer poly(vinylidene fluoride-co-hexafluoropropylene) (PVDF-HFP) membrane with a highly porous and lamellar structure through a combination of evaporation-induced phase separation and selective solvent etching methods. The developed membrane was capable of a greater electrolyte uptake and excellent electrolyte retention owing to its superior electrolyte wettability and highly porous structure, thereby offering better electrochemical performance than that of a commercial polyolefin separator (Celgard). Moreover, benefiting from the layered configuration, the tensile strength of the membrane could reach 13.5 MPa, which is close to the mechanical strength of the Celgard type along the transversal direction. The elaborate design of the multilayered structure allowed for the fabrication of a new class of thin separators with significant mechanical and electrochemical performance improvements. The thermal stability of the developed membranes was also better than that of the Celgard separator. Considering the electrochemical properties, the LIB cell adopting the three-layer membrane exhibited the highest capacity, the best rate capability, and compatible cycling stability, making it a preferable thin separator for high-power (fast charging) LIBs with superior cycle performance and high safety.

From the eight papers in this Special Issue, readers can obtain a variety of information on the polymer electrolytes currently being studied. The importance of electrolyte membranes in electrochemical energy conversion systems has recently been emphasized, and many further studies are expected.
